# Native and invasive ants affect floral visits of pollinating honey bees in pumpkin flowers (*Cucurbita maxima*)

**DOI:** 10.1038/s41598-021-83902-w

**Published:** 2021-02-26

**Authors:** Anjana Pisharody Unni, Sajad Hussain Mir, T. P. Rajesh, U. Prashanth Ballullaya, Thomas Jose, Palatty Allesh Sinu

**Affiliations:** 1 Central University of Kerala, Periya, Kerala 671316 India; 2 Sher-E-Kashmir University of Agricultural Science and Technology of Kashmir, Wadura, Kashmir 193201 India; 3 Present Address: Max-Planck Institute for Chemical Ecology, Jena, Germany

**Keywords:** Agroecology, Ecosystem services, Tropical ecology

## Abstract

Global pollinator decline is a major concern. Several factors—climate change, land-use change, the reduction of flowers, pesticide use, and invasive species—have been suggested as the reasons. Despite being a potential reason, the effect of ants on flowers received less attention. The consequences of ants being attracted to nectar sources in plants vary depending upon factors like the nectar source's position, ants' identity, and other mutualists interacting with the plants. We studied the interaction between flower-visiting ants and pollinators in *Cucurbita maxima* and compared the competition exerted by native and invasive ants on its pollinators to examine the hypothesis that the invasive ants exacerbate more interference competition to pollinators than the native ants. We assessed the pollinator's choice, visitation rate, and time spent/visit on the flowers. Regardless of species and nativity, ants negatively influenced all the pollinator visitation traits, such as visitation rate and duration spent on flowers. The invasive ants exerted a higher interference competition on the pollinators than the native ants did. Despite performing pollination in flowers with generalist pollination syndrome, ants can threaten plant-pollinator mutualism in specialist plants like monoecious plants. A better understanding of factors influencing pollination will help in implementing better management practices.

## Introduction

Humans brought together what continents isolated. One very conspicuous evidence of this claim rests in the rearranged biota. With the age of exploration, migration, and commerce, the previously unlikely event of organisms crossing the biogeographical and ecological barriers became typical^1^. Sometimes the introduced species could become a threat for native flora, fauna, ecosystems, and human welfare^2^. Despite causing a direct effect on biodiversity, the invasive species pose deleterious consequences to the community structure and ecosystem^3,4^.

One of the most successful taxa that invaded islands, mainland, and continents worldwide is ants^5^. Their sociality and colony structure backing their success cause widespread damage in the introduced area^6^. They are known to crash the populations of native ants, other invertebrates, reptiles, birds, and mammals in invaded sites^6,7^. Managing invasive ants has become a vital conservation tool^8^. Invasive ants participate and disrupt a range of plant-animal interactions as well^9,10^. Although there are cases when invasive ant interactions contribute to plant fitness as well^11^, the cost–benefit balance of such interactions are subtle and often result in negative repercussions^12^.

It is widely believed that plants defend ants effectively on flowers either by producing ant deterrents in flowers or by distracting ants to extra-floral nectaries distributed on non-floral parts of plants^13–15^. Plants in over 100 families use extra-floral nectaries for defending ants on flowers^16^. However, growing evidence shows that ants are also exploring flowers of a range of flowering plants, and giving differential impacts to pollinators, pollination, and plant reproduction^17–26^. Nevertheless, ants are mainly regarded as floral nectar thieves and disruptors of plant-pollinator mutualism^19,20,27–31^.

Among ants, the invasive ants have been particularly demonstrated to have a disruptive effect on plant-pollinator mutualism^20,23,24,29–33^. Invasive ants, because aggressive and competent in locating carbohydrate-rich sources in a short period, can occupy more flowers in more numbers, which can significantly reduce diversity, frequency, and foraging time of legitimate pollinators on flowers^12,34–36^. While most of the nectar-thieving ants primarily engaged in foraging floral nectar, a few also have attempted to prey upon pollinators^18,20,27–29^. Although some studies suggest that the invasive ants protect some plants against herbivores, it is likely that their aggressiveness wards off some useful mutualists, too^29,35^. Although a few studies examined the effect of invasive ants on pollinators, a comparative study between native and invasive ants has not been done yet^12^. In this study, we have attempted to compare the effects of ants on pollinators and compare the competition exerted by non-native and native ants on the pollinators of *Cucurbita maxima*—a monoecious globally-important, pollination-deficient crop plant.

To understand the effects of ants on pollinators, we took three attributes of pollinators and observed how these were affected by flower-visiting ants (hereafter, floral ants). They were the pollinators’ choice to visit or not to visit the flowers, the frequency of pollinators' visits (visitation rate), and the time the pollinators spent inside the flowers. We expected the visitation rate and visit duration of pollinators to be negatively affected by ants' presence. Since invasive ants are known for displacing biodiversity and interrupting species interactions, we hypothesize that the invasive ants exert more pressure on plant-pollinator interaction than the native ants do. We considered the ant's ability to occupy the maximum number of flowers and maximize the number of individuals occupying a single flower as the parameters for assessing ants' exploitation competition on pollinators. Our expectation for this was that the flowers occupied by invasive ants in the population are higher than the flowers occupied by native ants. Since the plant in our study is monoecious, we also asked whether the ant-pollinator interaction was affected by flower sex.

## Material and methods

### Study site

Our study was conducted at Kasaragod, the northernmost district of Kerala state in the peninsular India. The district is located at 12.5° N 75.0° E with an average elevation of 19 m above sea level. The highest and lowest temperature is 37 °C and 17 °C respectively. The district enjoys a tropical and sub-tropical climate, with winter (January and February), hot summer (March–May), and two rotations of Monsoon, the Southwest Monsoon (June–September) and Northeast Monsoon (October to November), giving an average annual rainfall of approx. 2000 mm. The district exhibits a topographic division into three, the lowland bordering the sea, the mainlands, and the forest highlands on the eastern side. The coastal strips are sandy, the lowland has red ferruginous loam with a mixture of clay and sandy soil, and the highland region is laterite.

The study took place in seven independent farmer-managed vegetable gardens in Kasaragod. These sites stood 3–10 km apart. The farmers of these sites cultivate rice paddy as their main crop, sowing in June to August and harvesting in November to January months of the year. From November to April, they cultivate vegetables belong to Amaranthaceae, Cucurbitaceae, Malvaceae, Solanaceae, and Fabaceae families as smallholdings (approximately one-acre land).

### Study system

We took pumpkin (*Cucurbita maxima*) as our study system. It is a monoecious plant with separate staminate (male) and pistillate (female) flowers. Both flowers are borne on different nodes with a disproportionately high number of staminate flowers per field^37^. The staminate and pistillate flowers are large, showy yellow with abundant floral nectar—the pistillate flowers producing a slightly higher quantity of quality nectar^38^. We harvested nectar from pre-caged staminate (N = 14; 2 per site) and pistillate flowers (N = 14; 2 per site) soon after anthesis using Drummond micro-capillary tubes (10 μL) and estimated the sugar concentration using a hand-held refractometer.

A large ovary at the flower base and three stigma lobes inside the flower distinguishes the pistillate flower from the staminate flower. The staminate flower has its anthers united into a single long filament of about 10 cm. The pollen grains are large and sticky; thus, pollination is dependent on the animal pollinator. Flower longevity is one day. The flower opened between 0630 and 0700 h and remained open for about 6 h. Native honey bee, *Apis cerana,* is the primary pollinator of *C. maxima* in our study area^37^. Farmers raised plants from the seeds collected from their previous years, with "Ambili" being the predominant variety in field populations. Four to six seeds were sowed in a pit of about 60 cm diameter and 30–45 cm depth, mixing it with neem cake and cow dung. The field is irrigated daily morning using the traditional pot method or water hose^38^.

### Sampling method

Our study's observations were carried out from 28th December 2018 to 15th February 2019, at the peak season of pumpkin flowering. During the study days (44 days), the data on ant visits and pollinator visits were collected from 0700 to 0800 h. We counted a total of 1664 flowers from all seven sites together to record the number of pistillate and staminate flowers. From this, we studied the pollinator and ant activity on 348 randomly chosen flowers (206 ant-occupied flowers (93 staminate and 113 pistillate flowers) and 142 ant-unoccupied flowers (33 pistillate and 109 staminate flowers)) belong to 102 plants (14.57 ± 21 plants/site). To observe pollinator and ant activity, we spent 15 min per flower during the early hours of the anthesis and pollinators' peak activity (0700–0800 h). We observed the flowers in which ants were present and the flowers in which ants were absent in the fields. During this observation window, we assessed the visitation rate, the duration of the visit, and pollinators’ hovering activity. We observed the visitation rate as the number of times the pollinator entered the flower. We recorded the time of entry and exit of a pollinator, the duration the pollinator spent inside the flower in a single visit. This time was recorded using a stopwatch. In cases, when a large number of ants colonised the flower, the pollinators did not enter the reproductive part of the flower; the pollinator rather hovered around the flower or landed on the tip of petals for very less time (< 3 s). Such an activity of the pollinators was recorded as 'hovering' and not as a visit. In flowers occupied by ants, we collected the ants in 5 mL vials with ethanol and identified them later from the lab. Based on the identification and the invasive ant list^39^, we grouped ant-occupied flowers into native ant flowers and invasive ant flowers. We also counted the number of ants occupied  a single flower. Whenever the ant colony was too large in number, the flowers were cut open to facilitate counting the ant individuals.

### Data analysis

First, we examined the preference of pollinators and ants to flower sexes for foraging resources. The visitation rate of pollinators on ant-unoccupied staminate and pistillate flowers was compared to understand pollinators' general preference for flowers for foraging floral resources using a Generalized Linear Mixed Effect Model (GLMM). In the model, pollinators' visitation rate was fitted as the dependent variable, flower sex was fitted as the fixed variable, flower ID nested in plant ID and sites was fitted as a random factor, and negative binomial distribution as an error type. The overall ants' abundance on staminate and pistillate flowers was compared using another GLMM. We repeated this for native ants and invasive ants, separately. In the models, respective ants' abundance was fitted as response variable, flower type as fixed effect, flower ID nested in plant ID and sites as random factors and negative binomial distribution as the error type.

We took three main attributes of pollinators—flower choice (on a binary scale; ant-occupied flowers or ant-unoccupied flowers), visitation rate and visit duration—to study the effects ants have on flowers. The pollinators’ choice of visit or not visit the ant-occupied and ant-unoccupied flowers was tested using a generalized linear mixed model with binomial distribution as the error type, visit-no visit data of pollinators on flowers (binary response) as the dependent variable, flower type as the fixed factor, flower ID nested in plant ID and sites as random factors and logit as a link function. We studied the effect of flower type (ant-unoccupied and ant-occupied) on visitation rate and the time the pollinators spent in flowers using two generalized linear models with negative binomial distribution as an error type, flower type and flower sex (staminate and pistillate) as the fixed effects and visitation rate and time spent by the bees as the response variables. We studied the interactive effects of ant type (invasive/native), number of ants and flower sex on a) visitation rate and b) the time the pollinators spent on ant-occupied flowers using two GLMMs. In the models, the ant type, number of ants on flowers, and flower sex were the fixed factors, visitation rate and the time the pollinators spent on flowers were the response variables, flower ID nested in plants and sites were the random factors and negative binomial distribution as the error type. We also examined the effect of ant species on visitation rate and time the pollinators spent on flowers by constructing two other GLMMs. These overall models' significance was tested using Wald’s Chi-square test available in the R-package *car*. In the results, mean ± standard deviation and estimate  ± standard error are given throughout. All analyses were carried out using R version 3.5.2^40^.

## Results

The pistillate flowers produced an average of 5.2 μL (± 0.09) nectar and the staminate flowers produced an average of 2.8 μL (± 0.08) nectar immediately following the anthesis, which continued and maintained through the entire anthesis period. In non-harvested flowers, the accumulated nectar volume can go up to about 40 μL in pistillate flowers and 28 μL in staminate flowers (Sinu, personal observation). The quality of nectar produced by staminate flowers averaged 29.4% (± 1.05) sugar concentration by weight and 31.1% (± 2%) in pistillate flowers.

The native honey bee, *Apis cerana*, was the primary pollinator of pumpkin flowers (92% of the total sample). In 8% of the flowers, another native bee species, *Apis dorsata*, also visited the flowers along with *A. cerana*. Since *A. dorsata* was a minority, we used pooled data of visitation characteristics of both the species in the analyses after realizing that the response of both the species of bees was the same towards the ants.

**Figure 1:**
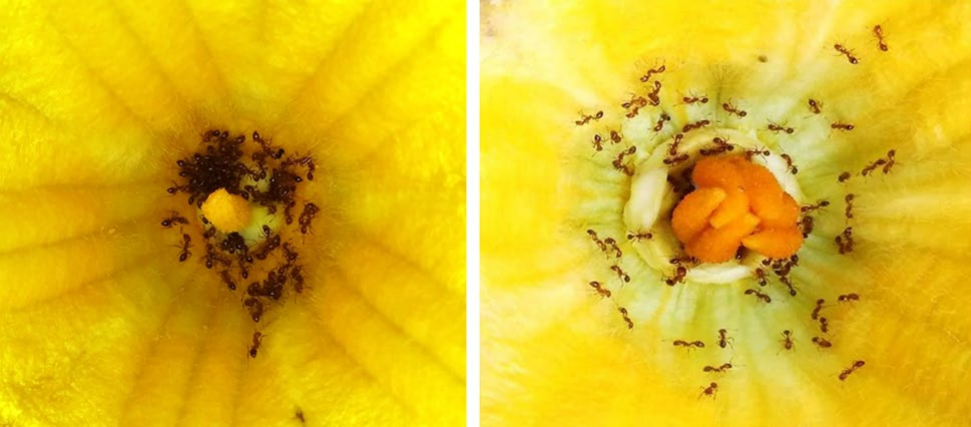
A staminate (male) and a pistillate (female) flower of pumpkin (*Cucurbita maxima*) occupied by the ants.

Nine species of ants visited the flowers of pumpkin as soon as the anthesis had occurred (Fig. 1). Four were non-native—*Anoplolepis gracilipes* (Smith, F.), *Paratrechina longicornis* (Latreille), *Solenopsis geminata* (Fabricius) and *Tapinoma melanocephalum* (Fabricius)—and five—*Camponotus variegatus*, *Polyrhachis excercita*, *Monomorium* sp, *Diacamma ceylonense*, *Tetramorium mixtum*—were native species. Ant species' composition was different among sites, but at least one of the native and invasive ant species was present in all the seven studied areas. The fire ant, *S. geminata,* occupied the highest number on a single flower, a maximum of 137 individuals. The black crazy ant, *Paratrechina longicornis*, occupied maximum number of flowers (53 flowers), followed by the yellow crazy ant*, Anoplolepis gracilipes* (49 flowers). The number of flowers having *Diacamma ceylonense* (N = 4), *Monomorium* sp (N = 1), *T. melanocephalum* (N = 7) and *P. excercita* (N = 1) was meager in the sample, so not used in the analyses.

On ant-unoccupied flowers, the staminate flowers received less pollinator visits (3.17 ± 3.8) than the pistillate flowers (7.4 ± 8.7; − 0.34 ± 0.13 z = − 2.58, *p* = 0.009). The average number of native ants on the flowers (10.4 ± 11.4) was significantly lesser than the average number of invasive ants on the flowers (32.6 ± 36.6; est ± se = − 0.84 ± 0.15, z = − 5.68, *p* < 0.00005). The number of overall foraging ants (− 0.21 ± 0.12, z = − 1.78, *p* = 0.07), invasive ants (− 0.23 ± 0.14, z = − 1.63, *p* = 0.10) and native ants (− 0.15 ± 0.12, z = − 1.19, *p* = 0.2) on staminate flowers was lesser than on pistillate flowers, but the difference was marginally or not significant.

**Figure 2:**
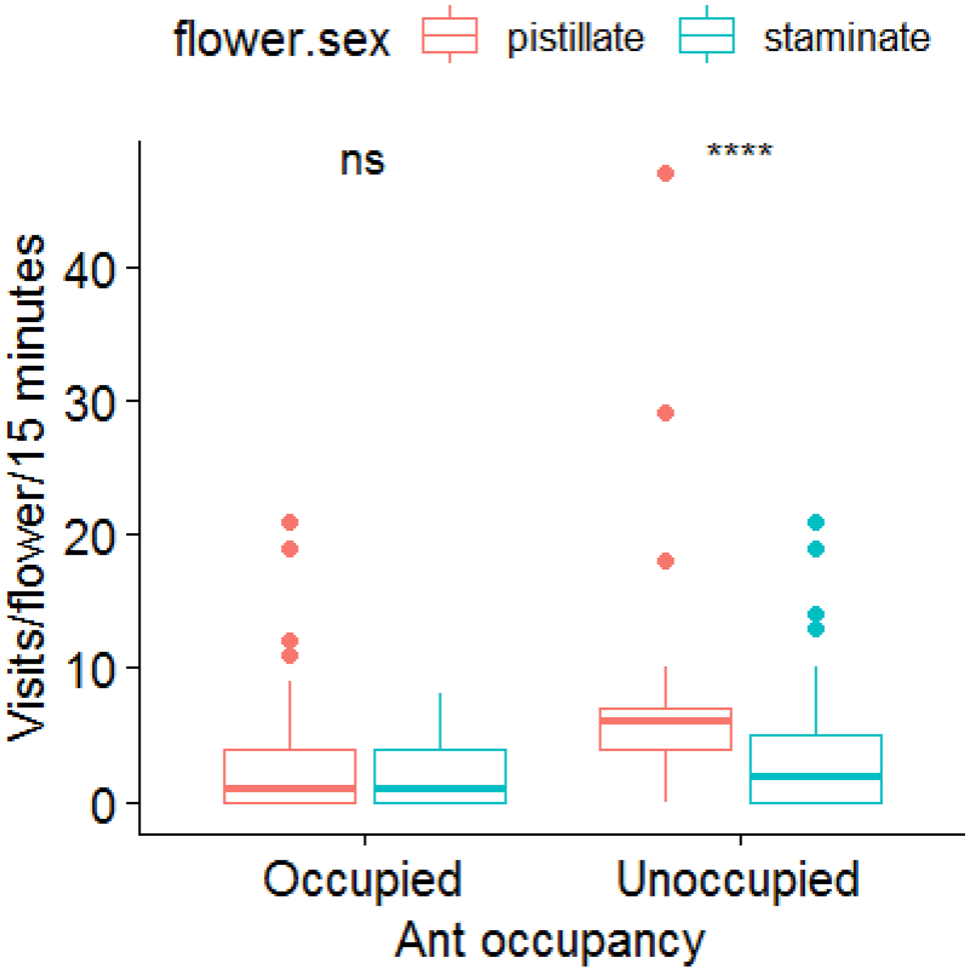
Visitation rate of pollinators to ant-occupied and ant-unoccupied staminate and pistillate flowers of *Cucurbita maxima*. ****p = 0.009.

The pollinators preferred to visit ant-unoccupied flowers (75%) over ant-occupied flowers (62%; 1.94 ± 0.6, z = 3.25, *p* = 0.001). The visitation rate of pollinators on ant-unoccupied flowers (4.14 ± 5.6) was significantly higher than on the ant-occupied flowers (2.4 ± 3.08; 0.50 ± 0.09, z = 5.35, *p* < 0.00005) (Fig. 2). On ant flowers, the visitation rate of pollinators to the native ants- (2.43 ± 2.29) and invasive ants-occupied flowers (2.38 ± 3.34) was same (0.41 ± 0.25, z = 1.62, *p* = 0.10), but decreased on native ants-occupied flowers when their numbers increased on the flowers (− 0.11 ± 0.04, z = − 2.41, *p* = 0.01). However, the visitation rate of pollinators to the flowers varied with the ant species occupied on the flowers (Wald’s χ^2^ = 18.75, df = 4, *p* = 0.0008) (Tables 1 and2). The pollinator visits also decreased with the number of ants occupied on flowers (− 0.04 ± 0.003, z = − 4.87, *p* < 0.00005). The response (visitation rate) of pollinators to the number of ants and ant type was not driven by flower sex (three-way interaction model: Wald’s χ^2^ = 0.93, df = 1, *p* = 0.33). It suggests that the pollinators, despite preferred pistillate flowers for foraging, absconded when the number of ants, though belonging to the native species group, was high on flowers (Table 2).

**Table 1:** Number of ants, visitation rate of pollinators and time spent per visit by the pollinators on flowers occuped by different species of ants.

Ant species	Nativity	Number of flowers	Number of ants/flower	Visitation rate	Number of visits	Time spent/visit
*Tetramorium mixtum*	Native	24	4.12 ± 2.51	3.21 ± 1.47	120	74.6 ± 25.9
*Camponotus variegatus*	Native	29	15.8 ± 12.6	2.1 ± 2.73	300	85.5 ± 68.5
*Anoplolepis gracilipes*	Invasive	49	27.22 ± 26.5	2.08 ± 2.43	180	71.07 ± 38
*Paratrechina longicornis*	Invasive	53	19.2 ± 25.22	3.47 ± 4.42	420	71.5 ± 72.8
*Solenopsis geminata*	Invasive	38	58.11 ± 48.8	1.47 ± 2.4	240	71.5 ± 46.6

**Table 2:** The GLMM models used to examine whether ant nativity (invasive vs. native), ant abundance and flower sex (pistillate vs. staminate) affect visitation rate and visit duration of pollinators on pumpkin flowers individually or in interaction among themselves.

Variable	Visitation rate			Visit duration		
	Estimate ± s.e	z-value	*p* value	Estimate ± s.e	z-value	*p* value
Flower sex	0.23 ± 0.17	1.32	0.18	0.04 ± 0.01	0.38	0.7
Number of ants (N.ants)	− 0.04 ± 0.008	− 4.87	< 0.0005	− 0.01 ± 0.002	− 6.88	< 0.0005
Ant nativity	0.41 ± 0.25	1.62	0.10	0.49 ± 0.19	2.59	0.009
Flower sex × N.ants	− 0.11 ± 0.11	− 0.98	0.32	− 0.02 ± 0.005	− 3.18	0.001
Flower sex × ant nativity	− 0.28 ± 0.32	− 0.87	0.38	− 0.58 ± 0.24	− 2.37	0.02
N.ants × Ant nativity	− 0.11 ± 0.04	− 2.41	0.01	− 0.09 ± 0.03	− 2.79	0.005
Flower sex × N.ants × Ant nativity	0.05 ± 0.05	0.96	0.33	0.11 ± 0.04	2.5	0.01

Negative binomial distribution fitted as error type in the models.

On ant-unoccupied flowers, the pollinators spent more time on pistillate flowers (96 ± 99 s/visit) than on staminate flowers (79 ± 89 s/visit; 0.14 ± 0.01, z = 7.16, *p* < 0.00005). The pollinators spent more time on ant-unoccupied flowers (85.02 ± 93.42 s/ visit) than on ant-occupied flowers for foraging (72.8 ± 58.3 s/ visits; 0.13 ± 0.06, z = 1.98, *p* = 0.04). On ant flowers, the foraging time of pollinators a) decreased with the number of ants on flowers (− 0.02 ± 0.002, z = − 6.39, *p* < 0.00005), b) not affected by the nativity of ant species (0.22 ± 0.13, z = 1.77, *p* = 0.07; 80.5 ± 54.4 s/visit for native ants- and 69.4 ± 59.6 s/ visit for invasive ants-occupied flowers), c) but decreased on native ants-occupied flowers when their numbers increased on them (− 0.09 ± 0.03, z = − 2.79, *p* = 0.005). The three-way interaction by the number of ants, ant type (native vs. invasive), and flower sex predicted the foraging duration of pollinators on flowers. (Table 2).

## Discussion

Ants have differential effects on plant-pollinator interaction^12,18–25^. Though a few studies demonstrate ants’ engagement in pollination^25,26^, ants have generally been regarded as a threat for plant-pollinator mutualism^19,20,23,24,27–31^. There are a handful of studies exploring the impacts of invasive ants on pollinators, however, a comparison of invasive and native ants concerning their interaction with floral visitors are scarce^12^.

In this study, we attempted to explore the effects of ants on pollinators and compare these effects in cases of invasive and native ants. The study found that ants, regardless of their nativity, gave a competitive pressure for honeybees—the sole pollinators—in pumpkin flowers. The visitation rate and the duration they spent foraging floral resources were significantly low for ant-occupied flowers. Although the bees tend to spend more time on native-ant-occupied flowers, when the number of ants is high, avoid such flowers.

With its showy flowers with a reasonable amount of floral rewards in the form of both pollen and nectar, pumpkin is a noteworthy lure for both ants and bees. We found that ants occupied more than 75% of the flowers during the early hours of anthesis in different fields. The ratio of staminate flowers to pistillate flowers counted in our study agrees that the number of staminate flowers is mostly higher in a field^37,41,42^. The ants preferred pistillate flowers over staminate flowers for foraging nectar, although they were less in field populations. They even tended to be crowded on pistillate flowers than on staminate flowers. The relatively good amount of nectar in pistillate flowers might be explaining the differential level of attraction of ants to flower sexes.

Lach^32^ defined interference and exploitation competition in the context of floral ants and pollinators. Exploitation competition is considered dependent on how much a competitor can access and utilize a resource. In our case, we thought the ant's ability to occupy the maximum number of flowers and crowding a single flower as the parameters for assessing the exploitation competition. Interference competition is the efficiency with which one competitor ward off the other.

Ants generally repel pollinators by their aggressive nature^43^. Sometimes, the pollinator might choose not to enter a flower due to ants^20^. The scent of ants negatively influences the choice of pollinator’s entry into flower^19^. Our study found that the pollinator entry was significantly lower in the ant-occupied flowers. We also observed several hovering cases, wherein the bee hovered around the flowers or landed on petals but flew off. In such cases, the bees avoided contact with ants and did not touch the flower's reproductive structures, which may be a massive loss for the plant as pollination is likely to be directly affected. The pollination efficiency was also significantly lowered in ant-occupied flowers^20^. Although there were cases of predation attack by yellow crazy ants previously^20^, we found the native ant, *Diacamma ceylonense* was opportunistically preying upon honey bees in the present study (pers. observ.). The duration of pollinators’ visit was also significantly higher in ant-unoccupied flowers over ant-occupied flowers. This indicates direct interference competition that ants offer to the pollinators.

Although bees did not distinguish ants on their nativity while venturing flowers occupied by ants, bothered by the ant species occupied on the flowers. Although our previous study showed that yellow crazy ant could be a major threat to the foraging bees, this study showed that *Solenopsis geminata* (invasive ant), *Tetramorium mixtum* and *Camponotus variegatus* (native ant) could be the worst competitors for foraging bees on flowers*.* Although other ant species occupied flowers, only five species frequently encountered on the flowers.

There are several cases of ant-pollinated plants. In the Western Ghats, ants were recently reported as a pollinator of *Syzigium occidentale* inflorescence^26^. In the Atlantic rainforest system, ants are a major pollinator of aggregated inflorescence^44^. Although ant-visited or pollinated flowers are not rare in tropics^18,25,45^, they are mostly bisexual and have either generalist pollination syndrome or have no bee pollinators on them. We found some amount of pollen sticking onto the ants during our observation. However, as other functional traits of pumpkin flowers—monoecy and highly skewed male–female ratio in populations—did not resonate with the characters of ant pollinated flowers^20,46^, ants might not play a major role in the pollination of pumpkin flowers. Additionally, exposing flowers to ant body secretions could deter pollen viability^17^, which, however, is not universal for all ant species^47^. While they feed nectar, ants sometimes damage the pistil base affecting the seed set^38^. Pumpkin is a major floral resource for specialist bees, including honey bees, bumblebees, and squash bees in different parts of the world, which perform cross-pollination in their monoecious flowers. Because they are monoecious, the visitors should travel from staminate to pistillate flowers for effecting pollination. It is doubtful that the ants switch visits between staminate and pistillate flowers in pumpkin fields as staminate flowers are abundant and pistillate flowers are not necessarily to be closer to staminate flowers. Although not investigated on all the nine species of ants in this study, Sinu et al.^20^ found that none of the *A. gracilipes-*only visited flowers sets fruits.

The efficiency with which ants used up resources determined the exploitation competition given by ants to the pollinators^32^. The main parameters of the ants that we used to measure this were the number of individuals that occupy a single flower and the number of flowers occupied by a single species of ant. *Solenopsis geminata* was found with the highest number of individuals per single flower. Although not particularly common for *S. geminata,* the mass recruitment is a key character of invasive ants^36^. Similarly, in general, invasive ants had a higher number of individuals recruited to a single flower than the native ants. The black crazy ant, *P. longicornis* occupied the highest number of flowers. This implies that they are highly efficient in locating resources. *P. longicornis* has a complicated modular recruitment system and polydymous nesting^36,48^. This minimizes the distance between the nest and food resource and increases their foraging efficiency. The other globally-important invasive species colonized in pumpkin flowers*, A. gracilipes*, is also known to make supercolonies and exploit resources quickly^49^. Thus, invasive ants exerted a higher exploitation competition on pollinators than native ants. Both the visitation frequency and the duration of visits decreased with the increasing number of ants. Therefore, even a native ant, when recruited in more numbers to the flowers, can also influence the pollinators' visitation attributes. The interference competition given by invasive and native ants seemed to be similar. Bees seemed not particularly showed any discretion between the scents of an invasive ant occupied and native ant occupied flower.

Our study further confirms the adverse effects the ants have on pollinators. However, such a generalization is challenging as the interactions between ants, plants, other herbivores, and pollinators are quite complicated^18,24^. For instance, Gonzálvez et al.^18^ showed that the native ants on flowers defend inefficient pollinators, but unaffected the visits of efficient pollinators. Unlike many previous studies, we compared the competition given by invasive ants and native ants on the pollinators. The invasive ants do most of the exploitive competition as they have their supercolonies and huge population to their advantage. However, we found that any ant—invasive or native—can exhibit interference competition by being aggressive and preying upon the pollinators. But it is not possible to draw a fine border between these two competitions. Information on the effects of ants on pollinators will help manage pollinators in agricultural lands^12,50^.
